# Monitoring Keap1–Nrf2 interactions in single live cells

**DOI:** 10.1016/j.biotechadv.2014.03.004

**Published:** 2014-11-01

**Authors:** Liam Baird, Sam Swift, David Llères, Albena T. Dinkova-Kostova

**Affiliations:** a Jacqui Wood Cancer Centre, Division of Cancer Research, Medical Research Institute, University of Dundee, Dundee DD1 9SY Scotland, UK; b Microscopy Facility, College of Life Sciences, University of Dundee, Dundee DD1 5EH Scotland, UK; c Institute of Molecular Genetics of Montpellier, 34293 Montpellier Cedex 5, France; d Department of Pharmacology and Molecular Sciences, Johns Hopkins University School of Medicine, Baltimore, MD 21205, USA

**Keywords:** Cytoprotective enzymes, FLIM, FRET, Keap1, Nrf2, Sulforaphane

## Abstract

The transcription factor NF-E2 p45-related factor 2 (Nrf2) and its negative regulator Kelch-like ECH associated protein 1 (Keap1) control the expression of nearly 500 genes with diverse cytoprotective functions. Keap1, a substrate adaptor protein for Cullin3/Rbx1 ubiquitin ligase, normally continuously targets Nrf2 for degradation, but loses this ability in response to electrophiles and oxidants (termed inducers). Consequently, Nrf2 accumulates and activates transcription of its downstream target genes. Many inducers are phytochemicals, and cruciferous vegetables represent one of the richest sources of inducer activity among the most commonly used edible plants. Here we summarize the discovery of the isothiocyanate sulforaphane as a potent inducer which reacts with cysteine sensors of Keap1, leading to activation of Nrf2. We then describe the development of a quantitative Förster resonance energy transfer (FRET)-based methodology combined with multiphoton fluorescence lifetime imaging microscopy (FLIM) to investigate the interactions between Keap1 and Nrf2 in single live cells, and the effect of sulforaphane, and other cysteine-reactive inducers, on the dynamics of the Keap1–Nrf2 protein complex. We present the experimental evidence for the “cyclic sequential attachment and regeneration” or “conformation cycling” model of Keap1-mediated Nrf2 degradation. Finally, we discuss the implications of this mode of regulation of Nrf2 for achieving a fine balance under normal physiological conditions, and the consequences and mechanisms of disrupting this balance for tumor biology.

## Introduction

### The Keap1/Nrf2 pathway

Cells have evolved multiple mechanisms to protect themselves under conditions of stress. One major protective mechanism comprises a network of functionally diverse inducible proteins, such as NAD(P)H:quinone oxidoreductase 1 (NQO1), heme oxygenase 1, glutathione transferases (GSTs), aldo-keto reductases, γ-glutamylcysteine ligase, thioredoxin, and thioredoxin reductase. The gene expression of these proteins is regulated by transcription factor NF-E2 p45-related factor 2 (Nrf2, gene name *NFE2L2*) (Itoh et al., 1997). Under homeostatic (basal) conditions, Nrf2 binds to its major negative regulator, Kelch-like ECH associated protein 1 (Keap1) (Itoh et al., 1999), which forms a RING E3-ubiquitin ligase with Cullin (Cul)3/Rbx1 and continuously targets the transcription factor for ubiquitination and proteasomal degradation (Cullinan et al., 2004, Kobayashi et al., 2004, Zhang et al., 2004). In response to electrophiles and oxidants (termed inducers), which recognize and chemically modify specific cysteine residues of Keap1, ubiquitination of Nrf2 is inhibited (McMahon et al., 2010). Consequently, Nrf2 accumulates and activates transcription, ultimately leading to enhanced expression of nearly 500 genes encoding drug-metabolizing, antioxidant, and anti-inflammatory proteins as well as enzymes involved in intermediary metabolism (Baird and Dinkova-Kostova, 2011, Hayes et al., 2010, Kensler et al., 2007, Malhotra et al., 2010, Mitsuishi et al., 2012a, Mitsuishi et al., 2012b, Singh et al., 2013). The coordinate expression of Nrf2 target genes results in enhanced protection against various types of stresses and restores homeostasis.

**Fig. 1:**
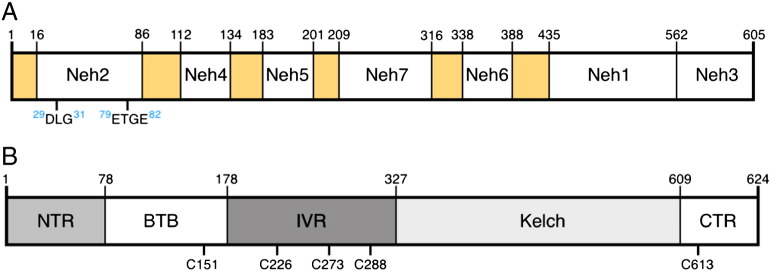
Domain structure of Nrf2 (A) and Keap1 (B). (A) In Nrf2, the positions of the seven functional domains Neh1-7 are shown. Neh1 contains the DNA binding and heterodimerization domain. The Neh2 domain is the main negative regulatory domain of Nrf2 through which it binds to Keap1 via the DLG and ETGE motifs. The Neh3, Neh4 and Neh5 domains bind to the transcriptional co-activators CHD6 and CBP. The Keap1-independent degradation of Nrf2 is mediated through the Neh6 domain. Nrf2 binds to the retinoid X receptor alpha (RXRα) through the Neh7 domain. (B) In Keap1, five functional domains are shown. Keap1 dimerization and Cul3 binding are mediated by the BTB domain. The intervening region (IVR) contains a number of reactive cysteine residues through which Nrf2 activity is regulated, including C226, C273 and C288. The Kelch domain forms a 6-bladed β-propeller structure through which Keap1 binds to the Neh2 domain of Nrf2, and to the KIR domain of p62, among other proteins.

Nrf2 is a 605 aa protein which has seven functional domains named Neh1-7 (Nrf2–ECH homology) (Fig. 1A) (Itoh et al., 1999). Neh1 contains the bZip DNA binding and heterodimerization domain through which Nrf2 forms a heterodimer with a small Maf transcription factor. The Nrf2/small Maf heterodimer binds to the antioxidant response element (ARE), also known as the electrophile response element (EpRE), the upstream regulatory sequence found in the promoter of cytoprotective genes. The Neh2 domain is a negative regulatory domain through which Nrf2 binds to Keap1. The Neh3 domain binds to the chromo-ATPase/helicase DNA binding protein family member CHD6, a transcriptional co-activator (Nioi et al., 2005). The Neh4 and Neh5 domains act synergistically to bind CBP, another transcriptional co-activator (Katoh et al., 2001). The Neh6 domain represents a second negative regulatory domain which mediates Keap1-independent degradation of Nrf2 (Chowdhry et al., 2013, McMahon et al., 2004, Rada et al., 2012). A third negative regulatory region is the Neh7 domain, through which Nrf2 interacts with the retinoid X receptor alpha (RXRα) (Wang et al., 2013).

Keap1 is a 624 aa multi-domain protein (Fig. 1B) which contains: (i) an N-terminal region (NTR, amino acids 1–60), (ii) a BTB domain (amino acids 61–179), through which Keap1 forms a homodimer and also interacts with Cul3, (iii) an intervening region (IVR, amino acids 180–314), which is especially cysteine-rich and contains 8 cysteine residues among its 134 amino acids, (iv) a Kelch domain, comprising six Kelch motifs (amino acids 315–359, 361–410, 412–457, 459–504, 506–551, and 553–598), through which Keap1 binds Nrf2, and (v) a C-terminal region (CTR, amino acids 599–624). Monomeric Nrf2 binds to dimeric Keap1 via two motifs residing in the N-terminal Neh2 domain of the transcription factor, the “DLG” and the “ETGE” motif (Fig. 1A), whereby the affinity for the ETGE motif is 200-fold greater than for the DLG (McMahon et al., 2006, Tong et al., 2006a). These motifs form β-turn structures which bind via electrostatic interactions between their acidic aspartate and glutamate residues with arginine residues 380, 415, and 483 in the Keap1 Kelch domain. Critically, both motifs are required for Keap1-mediated ubiquitination of Nrf2 by Cul3–Rbx1 (McMahon et al., 2006).

### Discovery of sulforaphane as an inducer of cytoprotective enzymes

**Fig. 2:**
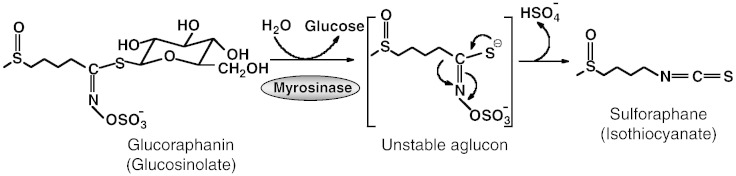
The myrosinase reaction. In the intact plant, the isothiocyanate sulforaphane is present as an inert precursor, the glucosinolate glucoraphanin. The hydrolysis of glucoraphanin is catalyzed by myrosinase which normally is compartmentalized in adjacent plant cells. Enzyme and substrate come in contact upon plant injury. As a result of the myrosinase-catalyzed hydrolysis, an unstable aglucone is formed first, and glucose is liberated. Depending on the reaction conditions, a series of final products can result, and the isothiocyanate sulforaphane represents one major product.

Sulforaphane [1-isothiocyanato-(4*R*)-(methylsulfinyl)butane] (Fig. 2) is an isothiocyanate which was isolated from broccoli extracts in Paul Talalay's laboratory as the principal inducer of the cytoprotective enzyme NQO1 by use of a quantitative bioassay-guided fractionation approach (Zhang et al., 1992). The isolation of sulforaphane was consequent to the finding that extracts of broccoli (*Brassica oleracea italica*) represent one of the richest sources of inducer activity among a large series of extracts prepared from the most commonly used edible plants that belong to 10 different plant families covering almost the entire spectrum of vegetables consumed in Europe and the USA (Prochaska et al., 1992).

In the intact plant, sulforaphane is present as a precursor, the glucosinolate glucoraphanin (Fig. 2) (Fahey et al., 2001, Talalay and Fahey, 2001). The same plant also contains a β-thioglucosidase enzyme, known as myrosinase (EC 3.2.1.147) which, under physiological conditions, is physically separated from its glucosinolate substrate. However, upon damage of the plant tissue such as injury or chewing, enzyme and substrate come in contact, resulting in highly efficient hydrolysis of the relatively biologically inert glucosinolate to form a variety of reactive products with critical functions for plant defense (Halkier and Gershenzon, 2006). Isothiocyanates, such as sulforaphane, represent one of the major types of products of the myrosinase reaction, and are responsible for the majority of the biological activities that have been associated with glucosinolates.

The central carbon of the isothiocyanate (—N=C=S) group is electrophilic and reacts readily with sulfur-, nitrogen-, and oxygen-centered nucleophiles. Such nucleophiles are contained within amino acids; consequently, proteins and peptides are the major cellular targets of isothiocyanates [reviewed in (Mi et al., 2011, Zhang, 2012)]. In particular, cysteine residues with low *pK_a_* values are especially reactive with isothiocyanates as they exist as thiolate anions even at physiological pH, and are thus primed for nucleophilic attack on the electrophilic substrate. As Keap1 is equipped with reactive cysteine residues, it serves as the cellular sensor for electrophiles, including isothiocyanates. Indeed, by use of UV–VIS spectroscopy cysteine modifications within Keap1 were shown to occur when the recombinant murine protein was incubated with sulforaphane (Dinkova-Kostova et al., 2002). By use of mutagenesis analysis, Donna Zhang and Mark Hannink found that ectopically-expressed Keap1 in which C151 in the BTB domain was mutated to a Ser is able to repress Nrf2 even upon sulforaphane treatment, thus implicating C151 as one of the sites which was specifically responsive to sulforaphane (Zhang and Hannink, 2003). Michael McMahon and John Hayes confirmed C151 as a target for sulforaphane by use of the biotin-switch technique, and further demonstrated by performing molecular modeling and mutagenesis experiments that C151 is particularly highly reactive as it is spatially surrounded by basic amino acids (H129, K131, R135, K150, and H154) which facilitate electrophilic addition to C151; indeed a mutant of Keap1 in which K131, R135, and K150 were replaced by Met residues had a greatly reduced sensor activity (McMahon et al., 2010). A model by Simon Fourquet and Michel Toledano predicted that C151 is remotely positioned from both the BTB dimerization interface and Cul3, and also implicated the basic amino acid environment in the increased reactivity of this cysteine (Fourquet et al., 2010). Based on mutagenesis analysis, Aimee Eggler and Andy Mesecar proposed an alternative model whereby large residues at position 151 cause steric clashes that lead to alteration of the Keap1–Cul3 interaction, ultimately resulting in impaired ability of Keap1 to target Nrf2 for ubiquitination (Eggler et al., 2009). Mass-spectrometry approaches have shown that, depending on the experimental conditions, in addition to C151, sulforaphane can also modify other cysteines within Keap1, including cysteines residing in the Kelch domain (Eggler et al., 2007, Hong et al., 2005a, Hu et al., 2011).

Following its isolation, sulforaphane was evaluated for the ability to induce the Nrf2-dependent enzymes NQO1 and GST in vivo. The activities of NQO1 and GST were upregulated in liver, forestomach, glandular stomach, small intestine, and lung of mice (Zhang et al., 1992), and in liver, colon, and pancreas, and bladder of rats (Matusheski and Jeffery, 2001, Munday and Munday, 2004, Zhang et al., 2006) following oral administration of sulforaphane. In contrast to wild-type animals, feeding sulforaphane in the diet for 14 days at a dose of 3 μmol/g diet did not affect the activities of NQO1 and GST in the small intestine of Nrf2-deficient mice (McMahon et al., 2001), implicating the Keap1/Nrf2 pathway as the main target and mediator of the inducer activity of the isothiocyanate. These efficacy studies are supported by investigations testing the ability of sulforaphane to protect against disease, first in a model of mammary carcinogenesis in Sprague–Dawley rats treated with single doses of the chemical carcinogen 9,10-dimethyl-1,2-benzanthracene (DMBA) (Zhang et al., 1994). It was found that oral administration of sulforaphane at doses of 75 or 150 μmol per day for 5 days, namely 3 days before, the day of, and the day after carcinogen exposure, decreased the incidence, multiplicity, and weight of the tumors. Since this initial animal experiment, sulforaphane has been used and shown to be an effective protective agent in numerous preclinical models of gastric, intestinal, prostatic, pulmonary, cutaneous and bladder cancers in animals, and in xenograft models of human tumors [reviewed in (Dinkova-Kostova, 2013)]. Furthermore, sulforaphane- or glucoraphanin-rich broccoli preparations have been and currently are in several human studies, ranging from healthy human subjects to populations at high risk for developing disease conditions [reviewed in (Dinkova-Kostova and Kostov, 2012) and registered at clinicaltrials.gov].

## Measuring protein–protein interactions with fluorescence lifetime imaging microscopy

### Resolution limits in conventional optical microscopy

Fluorescence microscopy is a powerful technique that has become central in the study of the structure and function of biological specimens. This is due in large part to its specificity and versatility. Although an understanding of structure has proved an important tool in understanding function while offering a mechanism to interrogate cells in the living state, conventional approaches are limited on the lateral axis to approximately half the wavelength of emitted light (for reviews see Stephens and Allan, 2003 and Lichtman and Conchello, 2005). Biological processes typically involve the action and regulation of multiprotein complexes and hence a key goal in most areas of cell biology is the characterization of the protein components within multi-subunit complexes through the reliable identification of specific protein interaction partners and the study of their dynamics. This has necessitated an approach that provides spatial information at an order of magnitude greater than can be achieved with standard imaging approaches (Chusainow et al., 2005). Indeed, to provide evidence of protein–protein interaction, it is required to measure the proximity of proteins with a resolution from 1 to 10 nm. This is simply not possible by using conventional fluorescence microscopy where the resolution of the light microscope is limited to ~ 250 nm laterally and ~ 500 nm axially. This limitation imposed by the visible light resolution can be overcome by the Förster resonance energy transfer (FRET) technique (Herman, 1989).

**Fig. 3:**
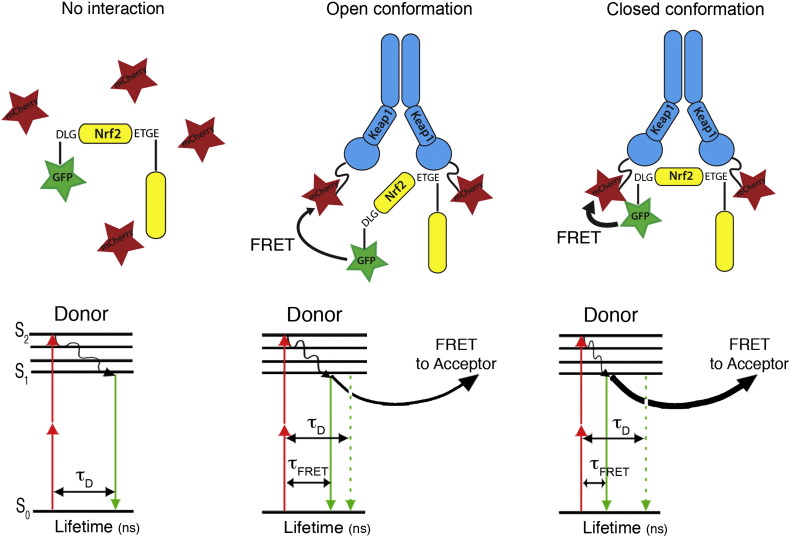
Detection of Förster resonance energy transfer (FRET) by fluorescence lifetime imaging microscopy (FLIM) between EGFP–Nrf2 and Keap1–mCherry fusion proteins. (Left) EGFP–Nrf2 and free mCherry fluorescent proteins do not interact (left, top). On absorbing 2-photons excitation light, the donor fluorophore changes from ground state (S_0_) to the excited state (S_2_), as illustrated in the simplified Jablonski energy-level diagram (left, bottom). This is followed by emission of a photon (fluorescence) during the next few nanoseconds (τ_D_). (Middle) EGFP–Nrf2 interacts with one monomer of the Keap1–mCherry dimer, and forms a Keap1–Nrf2 complex in an “open conformation” (middle, top). The two fluorescent fusion proteins interact, illustrating the effect of energy transfer on donor fluorescence lifetime. As the Jablonski diagram shows (middle, bottom), deactivation from the donor excited state can occur either by fluorescence (downward-pointing arrow), or through the radiationless transfer of energy to the acceptor by FRET. The occurrence of FRET is detectable by a decrease in the donor fluorescence lifetime (τ_FRET_). (Right) EGFP–Nrf2 interacts with both monomers of the Keap1–mCherry dimer and forms a Keap1–Nrf2 complex in a “closed conformation” (right, top). As illustrated by the thicker black arrow the non-radiative transfer of energy to the acceptor is stronger due to the closer proximity between EGFP–Nrf2 and Keap1–mCherry proteins. Consequently, a more drastic decrease in the donor fluorescence lifetime (τ_FRET_) is measured (right, bottom).

Förster resonance energy transfer (FRET) is the non-radiative transfer of excited-state energy from one molecule (named the donor) to another nearby molecule (the acceptor) (Fig. 3) (Clegg, 1996, Förster, 1965). FRET has proved a popular technique, increasing the spatial resolution of the fluorescence microscope to below 10 nm. FRET improves the spatial resolution because it relies on the close physical interaction of two fluorophores (the donor and the acceptor). Indeed, as the efficiency of energy transfer from the donor to the acceptor is dependent on the inverse of the sixth power of the distance separating them, FRET does not occur if the distance between these fluorophores exceeds 10 nm. Two further criteria must also be met: the emission spectrum of the donor must overlap the excitation spectrum of the acceptor, and the donor and acceptor must be appropriately orientated to allow energy transfer. Three methods for measuring FRET are routinely used: acceptor photobleaching, sensitized emission, and fluorescence lifetime imaging microscopy (Piston and Kremers, 2007, Sekar and Periasamy, 2003).

### Limitations of acceptor photobleaching and sensitized emission

It is worth noting the limitation of the first two of these approaches. In acceptor photobleaching, FRET can be measured by selectively photobleaching the acceptor molecule in order that energy from the donor can no longer be transferred to the acceptor. This leads to an increase in fluorescence intensity of the donor molecule. Although this is relatively straightforward in practice, not only can photobleaching of the acceptor cause photodamage to the sample, but in live-cell studies there is a significant risk that the FRET measurement will be invalidated by recovery of the acceptor fluorophore. In addition, this approach generally only measures FRET at a single predefined location, rather than at every pixel throughout the cell. As a result, acceptor photobleaching is not suitable for either time-lapse FRET measurements, or for detailed mapping of FRET locations at different sites within a cell. The second option, to measure FRET by sensitized emission, requires that the donor molecule is excited and fluorescence is measured in the acceptor channel only. In this case the donor molecule enters an excited state but transfers its energy to the acceptor rather than emitting light. This energy transfer elicits excitation of the acceptor molecule leading to fluorescence emission at a longer wavelength. The emission of light in the spectral range of the acceptor is then used as a measure of FRET. In practice, a fraction of this measured fluorescence will be due to direct excitation of the acceptor from the light used to excite the donor, and a fraction of measured fluorescence will be from fluorescent light coming from the donor. This cross-talk must be carefully measured and excluded, which can be difficult or, in the case of samples with high variation in each fluorophore concentration, most likely impossible to control (Swift and Trinkle-Mulcahy, 2004).

### Advantages of FLIM–FRET

**Fig. 4:**
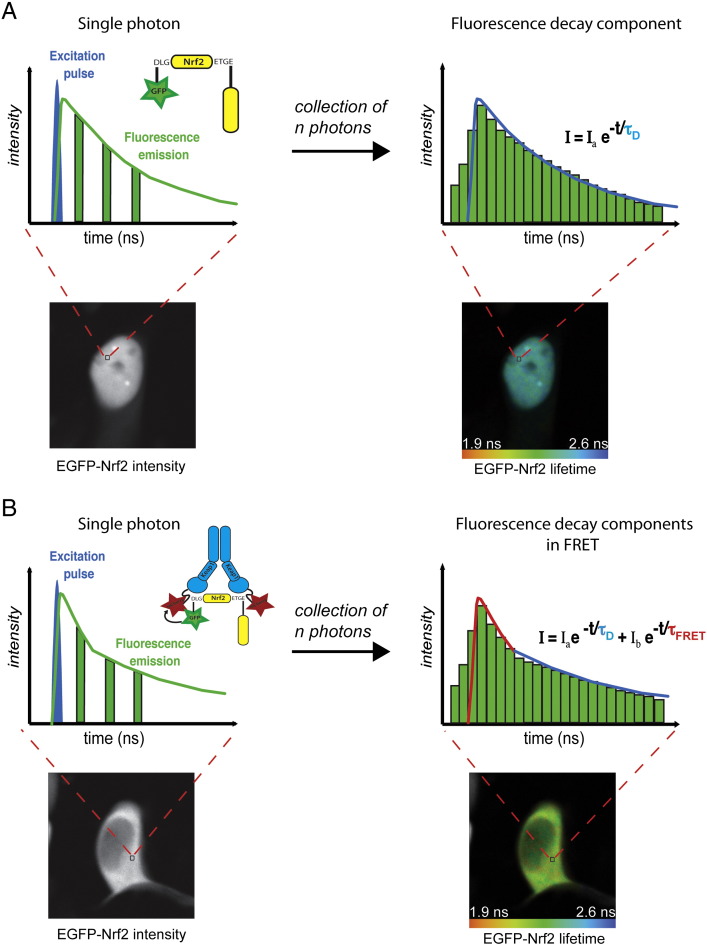
Time-correlated single photon counting (TCSPC) FLIM principle. The time-correlated single photon counting (TCSPC) FLIM system is based on the detection of single photons of a periodical light signal, followed by the measurement of the detection times of the individual photons, and the reconstruction of the waveform from the individual time measurements. For each pixel during the scanning acquisition time, when a single photon is detected from the donor EGFP–Nrf2 in the absence or in the presence of the acceptor Keap1–mCherry (A and B, respectively), the time of the corresponding detector pulse is measured. Each detection event is recorded in memory, associated with its specific detection time (left panels). Over time, the waveform of the optical pulse builds up, corresponding to a histogram presenting the number of photons recorded for each detection time interval (right panels). In the absence of FRET (A), a single exponential model is used to fit the experimental donor fluorescence decay. This analysis delivers the lifetime value τ_D_. In a FRET situation (B), a double exponential model can approximate the resulting donor fluorescence decay, with a slow lifetime component τ_D_ from the fraction of non-interacting EGFP–Nrf2 donor molecules (blue part of the fit) and a fast component from the fraction of interacting EGFP–Nrf2 donor molecules (red part of the fit). The composition of the donor decay function is written on the right. Double exponential decay analysis delivers the lifetimes, τ_D_ and τ_FRET_, and the intensity factors, a and b, of the two decay components. For each condition, a false-color image, displaying the distribution of the fluorescence lifetime for each pixel of the image, is shown.

The fluorescence lifetime imaging microscopy (FLIM) approach can be used to measure energy transfer and has the advantage of circumventing the fundamental problems associated with the two alternative methods previously described. When a fluorescent molecule absorbs a quantum of light, a valence electron is boosted into an excited state and returns to the ground state by: emitting a fluorescence photon, converting the energy internally, or transferring the energy to the environment, or a combination of some of these. In FLIM, the lifetime of the fluorescent event (i.e. the time it takes for the fluorophore to become excited and return to the ground state) is measured. The fluorescence lifetime of a fluorophore is an intrinsic property of the fluorophore that occurs on a nanosecond-time scale. Molecular excitation is stochastic, but the lifetime of a population of molecules can be plotted. If a large number of fluorescent molecules are excited by a short laser pulse, the time taken for fluorescence to decay can be plotted as a single exponential curve (Fig. 4A). Assuming that little or no energy is transferred to the environment, the fluorescence lifetime described by this decay curve is considered as the natural fluorescence lifetime. If energy is transferred to the environment, the fluorescence lifetime decreases. For almost all fluorophores, the rate of energy transfer from an electron in the excited state to the environment depends on the local chemical environment such as the concentration of ions, oxygen, pH value or the binding of proteins in a cell. FRET is an exceptionally strong quencher of fluorescence. Because there is a direct relation between the concentration of fluorescence quenchers and the fluorescence lifetime of the fluorophore, FLIM is particularly well suited for quantitative FRET analysis (Becker, 2012, Borst and Visser, 2010, Lakowicz, 2006).

Where FLIM–FRET proves most advantageous, is that it is generally insensitive to the relative concentrations of the fluorophores tagged to the proteins of interest and can be done in cell lines transiently expressing either one or both of the fluorophore-tagged proteins. The total decrease of the donor fluorescence lifetime depends both on the distance between the donor and acceptor and the fraction of interacting donor molecules. In FRET situations, a double exponential fluorescence lifetime model is used to describe the decay time of the donor molecule, with a slow lifetime component representing the fraction of non-interacting donor molecules and a fast lifetime component representing the fraction of interacting donor molecules (Fig. 4B). FLIM–FRET provides a unique measurement of the amount of FRET occurring for every pixel in an image, offering an exquisite level of spatial detail. Furthermore, it is not affected significantly by either the relative concentrations of the interacting proteins, or by their diffusion rates. FLIM–FRET can also provide additional information about the fraction of proteins engaged in interactions and their mean separation distance (Llères et al., 2007, Yasuda, 2006). For all these reasons, FLIM–FRET is now routinely used for dynamic measurements of protein–protein interactions and signaling pathways in living cells (Batisse et al., 2013, Ellis et al., 2008, Ems-McClung et al., 2013, Janes et al., 2009, Li et al., 2010).

## The cyclic sequential attachment and regeneration model of Keap1-mediated degradation of Nrf2

To gain a deeper understanding of the mechanism through which Nrf2 is targeted for ubiquitination by Keap1, we recently developed a FLIM–FRET-based system in cells expressing Keap1–mCherry and EGFP–Nrf2 fusion proteins (Baird et al., 2013). This approach allowed us to examine the dynamics of the Keap1: Nrf2 interaction in the endogenous environment of single live cells. FRET has previously been used to show that in the basal state, Nrf2 binds to Keap1 in the cytoplasm (Li et al., 2006). Recently, this approach has also been adapted for use in a high-throughput assay to identify direct inhibitors of the Keap1: Nrf2 interaction in vitro (Schaap et al., 2013). However, FRET has not previously been used to study the dynamism of the Keap1: Nrf2 interaction in live cells, or to determine how this interaction is affected by inducers.

**Fig. 5:**
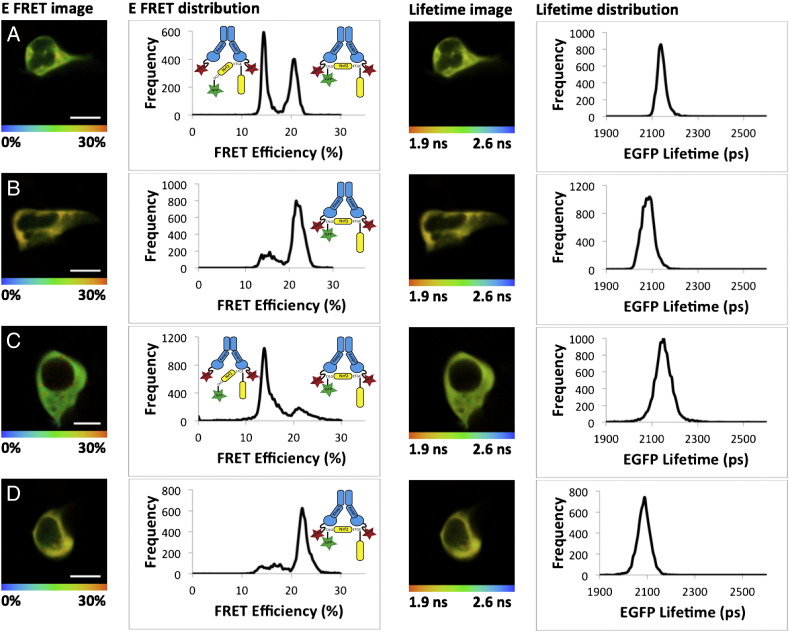
Inducers promote the formation of the closed conformation of the Keap1–Nrf2 complex. HEK293 cells were transfected with EGFP–Nrf2 + Keap1–mCherry and imaged 24 h later. Both the EGFP lifetime and FRET efficiency (E-FRET) were quantified in individual cells which were imaged twice, once in the basal state (A, C) and once again after 1-h treatment with either 5 μM sulforaphane (SFN) (B) or 10 μM STCA (D). The left column shows pictorial representations of the E-FRET where the color of the cell corresponds to the FRET efficiency according to the legend below the image, ranging from 0% to 30%. The second column shows the E-FRET from each pixel of the image plotted on a graph, with E-FRET on the x-axis and frequency on the y-axis. The graphs show that both SFN and STCA alter the FRET efficiency to favor the closed conformation (21% E-FRET population) of the Keap1–Nrf2 complex. This change can also be seen in the images in the first column, as both (B) and (D) contain more yellow and less green than (A) and (C). The third column shows a pictorial representation of the EGFP lifetime data from which the E-FRET data are derived. In these images, the color of the cell corresponds to the lifetime of EGFP, ranging from 1.9 ns to 2.6 ns as indicated on the legend below the image. The right column shows the lifetime data from each pixel of the image plotted on a graph, with lifetime on the x-axis and frequency on the y-axis. The graphs of the lifetime data show that in the presence of either SFN (B) or STCA (D), the lifetime of EGFP is reduced, manifesting as a shift in the EGFP lifetime to the left relative to the basal state shown in (A) and (C). This lifetime reduction is shown pictorially in the third column, where in the presence of either inducer the cells become yellow/orange and less green/yellow. Together these data show that in response to SFN or STCA, the lifetime of EGFP–Nrf2 is reduced, coupled with a change in E-FRET corresponding to an increase in the formation of the closed conformation of the Keap1–Nrf2 complex.

Using this system, we were able to calculate the FRET efficiency (E-FRET) within the Keap1–mCherry: EGFP–Nrf2 protein complex in such a way as to be able to “see” changes in the conformation of the protein complex. Surprisingly, the E-FRET distribution data revealed that there were two distinct FRET interaction populations, centered at 13% E-FRET and 21% E-FRET, respectively, implying that the Keap1: Nrf2 complex may be found in two different conformations in the basal state (Fig. 5A). In order to understand what these two FRET interactions represent, we generated a number of Nrf2 mutants which bound with either a reduced or increased affinity for Keap1 (Baird et al., 2013). Together, the mutant-derived data suggest that in the basal state the Keap1: Nrf2 complex is found in two distinct conformations: one in which only the high affinity “ETGE” motif of Nrf2 is bound to Keap1, representing the 13% E-FRET population (and termed the “open conformation”) and a second in which both the “DLG” and “ETGE” motif are bound to the Keap1 dimer, representing the 21% E-FRET population (and termed the “closed conformation”). Thus, the FLIM–FRET data suggested that the Keap1: Nrf2 complex in vivo exhibits a greater degree of dynamism than previously anticipated.

As changes in FRET can be directly related to changes in the conformation of the protein complex in which the fluorophores are found, we also analyzed the E-FRET of the Keap1: Nrf2 complex in the induced state. We used two inducers which differ in potencies and target different cysteines of Keap1, i.e., sulforaphane, which targets C151 (McMahon et al., 2010, Zhang and Hannink, 2003) and the sulfoxythiocarbamate STCA, which targets C273, C288, and C613, and is 25-times less potent than sulforaphane (Ahn et al., 2010). In the presence of sulforaphane (Fig. 5A,B) or STCA (Fig. 5C,D) the E-FRET was significantly altered, such that in the induced state, the Keap1: Nrf2 complex accumulated in the closed conformation (Fig. 5B,D), suggesting a functional change in the complex in response to inducers. Thus, both sulforaphane and STCA function to change the balance between the open and closed conformations of the Keap1: Nrf2 complex, whereby in the induced state, the closed conformation is favored.

The fact that inducers lead to a change in the conformation of the Keap1–Nrf2 complex suggests that the complex can “move” between the open and closed conformation states. In order to gain an understanding of how this dynamism is achieved, we manipulated the synthesis and degradation rates of Nrf2, through the use of the translation inhibitor cycloheximide (CHX) and the proteasome inhibitor MG132. Upon treating cells with either CHX or MG132 we observed a reduction in the abundance of the open conformation and a corresponding increase in the closed conformation (Baird et al., 2013).

**Fig. 6:**
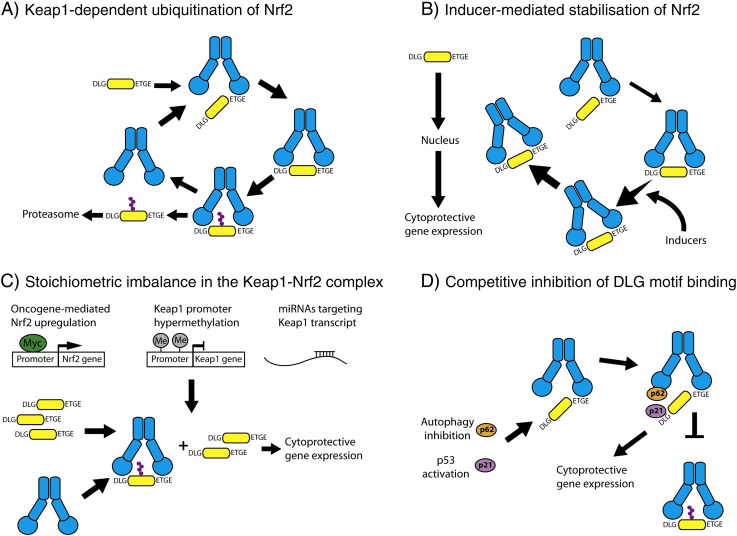
Nrf2-dependent regulation of cytoprotective gene expression. (A) The cyclic sequential attachment and regeneration model of Keap1-mediated degradation of Nrf2, with Keap1 in blue and Nrf2 in yellow. (B) Inducers produce a conformational change in Keap1 and thus uncouple the formation of the closed conformation of the complex from Nrf2 ubiquitination. This allows newly-translated Nrf2 to translocate to the nucleus and activate cytoprotective gene expression. (C) The Keap1-dependent cycle of Nrf2 degradation is finely balanced such that any increase in Nrf2 level, through enhanced transcription, or decrease in Keap1 level, by promoter hypermethylation or miRNA activity, leads to the saturation of Keap1 allowing the free Nrf2 to activate target gene expression. (D) When the Keap1–Nrf2 complex is in the open conformation, the Kelch domain of Keap1 and the DLG motif of Nrf2 are exposed and thus can bind to other proteins. This binding inhibits the formation of the closed conformation, and thus Nrf2 ubiquitination, and therefore allows other signaling pathways to regulate Nrf2 activity.

Together, these data indicate that the Keap1-mediated ubiquitination of Nrf2 follows a cycle as illustrated in Fig. 6A. In the basal state, newly-translated Nrf2 binds to one member of a free Keap1 dimer through its high affinity “ETGE” motif to form the open conformation. The existence of this conformation is supported by structural studies which have shown that the DLG motif binds to Keap1 in a distinct mode from the ETGE motif, with “fast on fast off” kinetics (Fukutomi et al., 2014). Subsequent to this, the low affinity “DLG” motif of Nrf2 binds to the other member of the Keap1 dimer to form the closed conformation. Once the two-site binding is achieved, the lysine residues in the α-helix between the “DLG” and “ETGE” motifs are in the correct orientation to be ubiquitinated by the Keap1-dependent E3-ligase (McMahon et al., 2006, Tong et al., 2006a, Tong et al., 2006b). Upon ubiquitination, Nrf2 is released from Keap1 and degraded by the proteasome, while the regenerated free Keap1 dimer is able to bind newly-translated Nrf2 allowing the cycle to continue. We call this model the “cyclic sequential attachment and regeneration” or “conformation cycling” model of Keap1-mediated Nrf2 degradation.

Our findings suggest that inducers function to stabilize Nrf2 by promoting the formation of the closed conformation of the Keap1–Nrf2 complex. This outcome seems counterintuitive as the closed conformation is associated with ubiquitination of Nrf2 in the basal state, yet with stabilization of Nrf2 in the induced state. Notably, these two ideas are not contradictory as the E-FRET data reflect the distance between Nrf2 and the Kelch domain of Keap1, but not the relative location of the ubiquitination machinery of the Keap1 complex. It has been shown that when inducers bind to the sensor cysteines of Keap1 they lead to a conformational change in the protein, but not to the dissociation of the Keap1–Nrf2 or the Keap1–Cul3 complex (Baird and Dinkova-Kostova, 2013, Dinkova-Kostova et al., 2005, Eggler et al., 2005, Li et al., 2012). The inducer-mediated conformational change in the Keap1 structure may alter the positioning of Nrf2 relative to the E2-ubiquitination machinery such that Nrf2 can no longer be ubiquitinated and/or degraded by the Keap1 complex (Fig. 6B). Thus, although in the induced state the Keap1: Nrf2 complex is still in the closed conformation, inducer-dependent conformational changes in Keap1 mean that Nrf2 can no longer be targeted for degradation. Consequently, Nrf2 is not released from Keap1, the free Keap1 dimer is not regenerated, and it can be proposed that, in the presence of electrophiles which target cysteine sensors of Keap1, Nrf2 acts as a “suicide” substrate to inactivate Keap1. The newly-translated Nrf2 is then unable to bind to Keap1 and thus is free to translocate to the nucleus and activate expression of cytoprotective genes. This mechanism explains why in the absence of new translation, Nrf2 is not stabilized by inducers, and Nrf2-dependent target genes are not upregulated (Kobayashi et al., 2006, Kwak et al., 2002, Sekhar et al., 2000, Shay et al., 2012).

## Implications of the cyclical nature of the Keap1-mediated degradation of Nrf2

### Mutations in either Keap1 or Nrf2 destroy two-site binding in tumors

The physiological importance of the two-site binding and formation of the closed conformation is illustrated by the mutation spectrum of Nrf2 in human cancer. Persistent upregulation of Nrf2-dependent genes is frequently exploited by cancer cells, and promotes their survival and resistance to chemotherapy and radiation therapy: mutations in *KEAP1* or *NRF2*, which abrogate formation of the complex and lead to Nrf2 accumulation and constitutive activation of the pathway, have been detected in several tumor types, including lung, liver, breast, kidney, ovarian, gall bladder and esophageal cancers (Abazeed et al., 2013, Guichard et al., 2012, Konstantinopoulos et al., 2011, Nioi and Nguyen, 2007, Ooi et al., 2013, Sato et al., 2013, Shibata et al., 2008a, Shibata et al., 2008b, Shibata et al., 2011). Comprehensive genomic characterization of squamous cell lung cancers (2012) has identified mutations in *NRF2*, *KEAP1*, or *CUL3* in 34% of 178 lung squamous cell carcinomas. According to the COSMIC database (http://cancer.sanger.ac.uk/cancergenome/projects/cosmic/), there have been 219 non-synonymous mutations found in the *NRF2* gene, of which 84% are found in or adjacent to either the “DLG” or “ETGE” motifs. In one study in which *NRF2* was found to be mutated in 12% of lung cancer patients, all 14 mutations identified were in either the “DLG” or “ETGE” motifs (Shibata et al., 2008b). These in vivo data highlight the fact that the formation of the closed conformation is of fundamental importance to the Keap1-mediated Nrf2 regulation.

Interestingly, a novel class of Keap1 cancer mutations have recently been identified which do not lead to the destruction of two-site binding. Instead, the newly termed “superbinder” Keap1 mutants bind to Nrf2 with a greater affinity than wild-type Keap1, yet are unable to inhibit Nrf2-mediated gene expression (Hast et al., 2014). These data from human tumors complement the conformation cycling model, and suggest that similar to small-molecule Nrf2 inducers, the “superbinder” mutants may lead to an accumulation of the Keap1: Nrf2 complex in the closed conformation, allowing newly-translated Nrf2 to bypass the “saturated” Keap1 and translocate into the nucleus.

### Stoichiometric imbalance

As the Keap1-dependent destruction of Nrf2 follows a cycle, the relative abundance of each protein will impact on the efficiency of the ubiquitination of Nrf2. For example, an increase in the amount of Keap1 relative to Nrf2 will tip the balance in favor of Nrf2 ubiquitination, as the increased abundance of Keap1 will be able to bind the cellular pool of Nrf2 more efficiently. Conversely, an increase in expression of Nrf2 relative to Keap1 will lead to the saturation of Keap1 binding and subsequent increase in the Nrf2 protein level (Fig. 6C). Indeed this has been shown to be the case using a mouse model to examine Nrf2 gene dosage effects (Suzuki et al., 2013). In this study, Suzuki et al. found that a reduction in the level of Nrf2 expression resulted in decreased Nrf2 activity in both the basal and induced states. Conversely, an upregulation in Nrf2 expression led to an increase in Nrf2 activity in both the basal and induced states, suggesting that Nrf2 activity is governed by a balance between protein synthesis and destruction due to the limited capacity of Keap1 to target Nrf2 for ubiquitination.

This Nrf2 gene dosage effect has important implications for human disease, particularly in cancer. For example, in a somatic copy number variation (SNCA) pan-cancer analysis, *NFE2L2*, the gene encoding Nrf2, was identified as one of the loci recurrently gained in human tumors (Zack et al., 2013). In addition, in a mouse model of pancreatic cancer it was shown that oncogenic K-Ras or B-Raf signaling resulted in a modest (< 2-fold) upregulation in Nrf2 expression, leading to an increase in Nrf2 protein level, and Nrf2-dependent gene transcription, which ultimately contributed to a cellular detoxification program that aided tumorigenesis in this model (DeNicola et al., 2011). Conversely, a reduction in the amount of Keap1 protein would have a similar effect, as it will become oversaturated with a basal level of Nrf2, resulting in the increased transcription of Nrf2-target genes in the absence of either Nrf2 upregulation or oxidative stress. One such mechanism through which the level of Keap1 expression may be reduced in the cell is through promoter hypermethylation. Indeed, in cell lines and tumors from patients with gliomas, lung cancer, colorectal cancer, prostate cancer, and head and neck cancer, hypermethylation of the Keap1 promoter has been observed (Hanada et al., 2012, Martinez et al., 2014, Muscarella et al., 2011, Wang et al., 2008, Zhang et al., 2010). This leads to a reduction in Keap1 expression, and in the case of prostate and colorectal cancer cells, an increase in the expression of Nrf2-target genes. Similarly, it has been shown that in breast cancer cells, the microRNA miR-200a is able to target a sequence in the 3′UTR of the Keap1 mRNA, leading to a reduction in the level of Keap1 mRNA and protein, and a concomitant increase in the level of Nrf2 and increased transcription of the Nrf2-target gene NQO1 (Eades et al., 2011). The physiological relevance of the stoichiometric imbalance of Keap1 and Nrf2 is well illustrated in non-small cell lung cancer (NSCLC). Tumors which are both positive for Nrf2 and show low or absent Keap1 expression are significantly associated with a reduced 5-year overall survival rate (Solis et al., 2010).

Interestingly, both cysteine-reactive inducers and non-canonical activation of Nrf2 may also impact the relative abundance of Keap1 upon pathway activation. It has previously been shown that in the presence of inducers, Keap1 and not Nrf2, becomes the substrate for ubiquitination (Hong et al., 2005b, Zhang et al., 2005). The ubiquitination of Keap1 leads to a reduction in its half-life in the induced state, and thus once modified by electrophiles, the relative abundance of Keap1 within the cell is reduced (Taguchi et al., 2012). Similarly, binding of p62 to Keap1 not only inhibits the formation of the closed conformation, but also targets Keap1 for degradation through the autophagy pathway (Bae et al., 2013, Taguchi et al., 2012). These data suggest that the rapid accumulation of Nrf2 in response to either electrophiles and oxidants, or other signaling pathways occurs due to the simultaneous inactivation of Keap1-mediated ubiquitination of Nrf2, and a decrease in the level of Keap1. Together, these complementary mechanisms facilitate the rapid and robust induction of cytoprotective gene expression.

### Protein competition

One consequence of the “conformation cycling” model is that the Keap1–Nrf2 complex must spend some of its time in the open conformation before progressing to the closed conformation. One simple question which arises from these data is: what is the biological function of the open conformation? We believe that the Keap1–Nrf2 complex forms the open conformation in order to provide an additional opportunity for other signaling pathways and cellular inputs to regulate Nrf2 activity by inhibiting the formation of the closed conformation (Fig. 6D). Thus competitive binding of other proteins to Keap1 provides another layer by which Nrf2-dependent gene expression can be regulated.

The first protein which was found to positively regulate Nrf2-dependent gene expression through competitive inhibition of two-site binding was the oncoprotein prothymosin α (Karapetian et al., 2005). Prothymosin α was found to contain an “ENGE” motif which is similar to the high affinity “ETGE” of Nrf2 (Padmanabhan et al., 2008). Through this motif, prothymosin α may be able to compete with the DLG motif of Nrf2 for binding to the Kelch domain of Keap1, leading to a loss of the formation of the closed conformation and the upregulation of Nrf2 target gene expression. A genomic screen for activators of Nrf2 found that p62 and DPP3 could also positively regulate Nrf2-dependent gene expression (Liu et al., 2007). Like prothymosin α, both p62 and DPP3 compete with Nrf2 for binding to Keap1, suggesting that this may be a common non-canonical mechanism by which cytoprotective gene expression can be regulated (Hast et al., 2013, Komatsu et al., 2010). Indeed, the list of proteins which are able to compete for binding with Nrf2 has increased, suggesting that multiple signaling pathways can converge on Nrf2 to regulate its activity (Table 1). This list includes human IKKβ, which has been shown to bind to Keap1 (Kim et al., 2010, Lee et al., 2009), although the consequences for Nrf2 activation have not been examined. In addition, one of the proteins which may inhibit the formation of the closed conformation, p21, binds not to the Kelch domain of Keap1, but directly to the DLG motif of Nrf2, and in doing so inhibits the two-site binding (Chen et al., 2009). This suggest that multiple mechanisms may be used to inhibit the formation of the closed conformation of the Keap1: Nrf2 complex, and thus inhibit Nrf2 ubiquitination.

**Table 1:** Examples of proteins which interact with Keap1 or Nrf2.

Protein	Binding partner	Motif	Reference
Prothymosin α	Keap1	ENGE	Karapetian et al. (2005) and Padmanabhan et al. (2008)
p21	Nrf2	KRR	Chen et al. (2009)
p62	Keap1	PSTGEL	Komatsu et al. (2010)
Palb2	Keap1	LDEETGE	Ma et al. (2012)
WTX	Keap1	ETGE	Camp et al. (2012)
PGAM5	Keap1	ESGE	Lo and Hannink, 2006, Lo and Hannink, 2008
DPP3	Keap1	ETGE	Hast et al. (2013)
IKKβ	Keap1	ETGE	Kim et al. (2010) and Lee et al. (2009)

The physiological consequences of this competitive binding mode of Nrf2 regulation are best understood in the case of p62. When autophagy is suppressed in the cell, p62 accumulates and binds to the Kelch domain of Keap1 through its KIR domain (Komatsu et al., 2010). Notably, by use of fluorescence polarization, it has been shown that a peptide based on the Keap1-binding motif of p62 interacts with the Kelch domain of Keap1, and curiously, the affinity of this interaction is 10-fold stronger when the p62-based peptide is phosphorylated (Hancock et al., 2012). In agreement, phosphorylation of S351 within the KIR domain by the mechanistic target of rapamycin complex 1 (mTORC1) kinase increases the affinity of p62 for Keap1 by 30-fold (Ichimura et al., 2013). During both mitophagy and xenophagy, p62 becomes phosphorylated at S351, and co-localizes with Keap1. This results in both the nuclear accumulation of Nrf2, and the upregulation of the Nrf2-target genes NQO1 and heme oxygenase 1 (Ichimura et al., 2013). Similarly, in mouse livers the suppression of autophagy leads to the accumulation of phospho-p62, its colocalization with Keap1, and the upregulation of Nrf2 target gene expression (Ichimura et al., 2013). Together, these data clearly demonstrate that non-canonical activation of Nrf2 through inhibition of the formation of the closed conformation of the Keap1: Nrf2 complex has important physiological consequences.

## Comparison of the “conformation cycling” model with the “hinge and latch” model

The “conformation cycling” model builds upon the previously proposed “two-site binding” or “hinge and latch” model of Keap1-mediated Nrf2 degradation (Fukutomi et al., 2014, Tong et al., 2006a, Tong et al., 2006b, Tong et al., 2007). The “hinge and latch” model postulates that, as both cancer-related mutations and non-canonical activators, such as p62, function to inhibit the two-site binding, inducers may function in the same way, and it has been proposed that in the presence of inducers, the low affinity DLG motif is released by Keap1. However, there are currently no experimental data which have demonstrated that the release of the DLG motif occurs. In addition, it is difficult to reconcile some published data with the “hinge and latch” model as it is currently understood. For example, McMahon et al. showed that if the low affinity DLG motif is mutated into an additional high affinity ETGE motif, Nrf2 can still be stabilized by the inducer sulforaphane (McMahon et al., 2006). This suggests that the release of the low affinity motif (which is not present in this mutant Nrf2) is not required for Nrf2 stabilization. In addition, isothermal titration calorimetry (ITC) experiments carried out with full-length Keap1 protein and the Neh2 domain of Nrf2 show that inducers do not lead to a reduction in the affinity of these two proteins, as would be predicted by the “hinge and latch” model (Eggler et al., 2005). It has also been shown that inducers, including sulforaphane, promote Nrf2 association with Keap1, rather than dissociation (Li et al., 2012). Importantly, the available structural data, as well as the cancer-related mutations and non-canonical stabilization of Nrf2 data support both the “hinge and latch” model and the “conformation cycling” model, and thus there are no published data which support the “hinge and latch” model that do not also support the “conformation cycling” model.

The structural data suggest that the ETGE and DLG motifs each bind to Keap1 in a distinct manner, and that the “rapid association–dissociation” nature of the DLG interaction means that it is sometimes bound to Keap1 and sometimes unbound (Fukutomi et al., 2014). This conforms exactly to the open and closed conformation of the Keap1: Nrf2 protein complex which we observed in our live-cell assay. Non-canonical activation of Nrf2 by proteins such as p62 support the significance of this finding, and suggest that Nrf2 forms the open conformation in order to allow multiple modes of regulation. The “hinge and latch” model is also supported by cancer-related mutations in Nrf2, where mutations in either the ETGE motif or the DLG motif are sufficient to inhibit two-site binding, resulting in Nrf2 stabilization. Again, these data equally support the “conformation cycling” model, as here two-site binding is also required for Nrf2 ubiquitination, so mutation of either binding motif will inhibit Nrf2 ubiquitination.

The “conformation cycling” model suggests that the inactivation of Keap1 by cysteine-reactive inducers is sufficient for Nrf2 stabilization. This explains why the presence of a second high affinity ETGE motif in Nrf2 still allows the protein to be stabilized, despite the fact that the “weak” binding motif (which is absent in this mutant) is not released. In addition, the “conformation cycling” model also explains why the isothermal calorimetry data showed no difference between the basal and induced state: namely because cysteine-reactive inducers do not affect the way by which Nrf2 binds to Keap1, but instead lead to the inactivation of Keap1-mediated Nrf2 ubiquitination.

One prediction that can be made based on the “conformation cycling” model is that in addition to mutations in human tumors leading to the destruction of the two-site binding, a subset of mutations in either Keap1 or Nrf2 may lead to an increase in the affinity of the two proteins. According to the cyclic model, an increased affinity of the Keap1: Nrf2 complex will reduce the rate of Keap1 regeneration, allowing newly-translated Nrf2 to translocate to the nucleus and activate cytoprotective gene expression. Interestingly, mutations have been identified from human tumors in both Keap1 and Nrf2 which increase the affinity of the mutant protein for its wild-type partner (Fukutomi et al., 2014, Hast et al., 2014). The mechanism by which these mutations may function is difficult to understand in light of the “hinge and latch” model, but is in complete agreement with the “conformation cycling” model. Thus, the “conformation cycling” model conforms to all of the available data which also support the “hinge and latch” model, and is also supported by data which until now, have not been fully explained.

## Conclusions

As the role of Nrf2 has expanded from cytoprotection to include regulating cell proliferation and metabolism, the inputs which activate Nrf2 activity have similarly diversified. Thus, Keap1-mediated Nrf2 activation is no longer limited to the sensing of exogenous electrophiles and oxidants, but can be regulated by p53 activation, autophagy defects, and changes in tricarboxylic acid (TCA) cycle enzyme activity (Adam et al., 2011, Bae et al., 2013, Komatsu et al., 2010, Ooi et al., 2011). The development of a FLIM–FRET system revealed that the Keap1: Nrf2 complex is able to integrate these diverse cellular signals to regulate Nrf2 activity through both the canonical as well as non-canonical pathways. This mechanism allows the cell to respond rapidly to multiple numbers of environmental and cellular changes through the upregulation of Nrf2-dependent gene transcription.

Nrf2 binds to the Keap1 dimer through two distinct motifs (McMahon et al., 2006, Tong et al., 2006a, Tong et al., 2006b). By use of Nrf2 mutants we found that the complex exists in two distinct conformations in the basal state, suggesting that the Keap1: Nrf2 complex is much more dynamic than previously anticipated. In the design of our fusion proteins, the relative positions of the fluorophores in the complex, coupled with the dual binding sites in Nrf2, facilitated the discovery of the conformational dynamism. These mechanistic considerations are not unique to the Keap1: Nrf2 interactions, and many signaling pathways are mediated by dynamic protein dimer complexes, including EGFR and Fbw7 (Dawson et al., 2005, Marianayagam et al., 2004, Welcker and Clurman, 2007). Therefore, an approach analogous to ours could be adapted to study other cellular pathways. Indeed, a dimer of the E3-ubiquitin ligase Fbw7 binds to a monomer of its target cyclin E through two distinct motifs, in a similar way to the Keap1: Nrf2 complex (Hao et al., 2007, Welcker and Clurman, 2007). Use of FLIM–FRET may reveal that the dynamic cyclic nature of the Keap1: Nrf2 complex is not limited to this pathway and may represent a more general mechanism through which other E3-ubiquitin ligases target their substrates for ubiquitination and degradation.
